# Higher-Risk Localized Prostate Cancer Is Associated With Increased Coronary Artery Calcium and Coronary Plaque Burden

**DOI:** 10.1016/j.jacadv.2025.101973

**Published:** 2025-07-08

**Authors:** Adithya K. Yadalam, Carlotta Onnis, Marly van Assen, Carlo N. DeCecco, Stephanie M. Cantu, Anant Mandawat, Sagar A. Patel

**Affiliations:** a Emory University School of Medicine, Atlanta, Georgia, USA; b Department of Radiology, University of Cagliari, Cagliari, Italy; c Winship Cancer Institute of Emory University, Atlanta, Georgia, USA

Androgen deprivation therapy (ADT), the cornerstone of systemic therapy for prostate cancer (PCa), is associated with a significantly increased risk for adverse cardiovascular events. As more men survive PCa with increasing ADT use, cardiovascular mortality has now surpassed cancer as the most common cause of death in men with PCa. Furthermore, emerging data have revealed that higher-risk PCa may be associated with higher cardiovascular disease risk.^1^ As higher-grade PCa is associated with increased systemic inflammation, which also drives coronary atherosclerosis, we hypothesize that higher-risk PCa may be associated with increased coronary artery calcium (CAC) and total plaque volume (TPV) burden, well-validated markers of cardiovascular risk.^2^^,^^3^

The study sample included men with newly diagnosed, localized PCa recruited to the RElugolix VErsus LeUprolide Cardiac Trial (REVELUTION-1, NCT05320406), a prospective trial investigating the impact of ADT on coronary atherosclerosis. Participants were recruited at Emory-affiliated hospitals in Atlanta, Georgia, between June 2022 and April 2024. Men aged ≥18 years with nonmetastatic, treatment-naive PCa undergoing curative-intent prostate radiotherapy with or without ADT were eligible for inclusion into REVELUTION-1. Exclusion criteria included metastatic PCa, prior ADT, prior cancer, or prior percutaneous coronary intervention or coronary artery bypass graft surgery. All patients underwent baseline electrocardiogram-gated cardiac computed tomography (Aquilion One GE CT750 HD, Canon) prior to starting cancer therapy. Calcium scoring was calculated in Agatston units (AU) by a board-certified cardiologist, and plaque volumes were estimated using a commercially available software service (HeartFlow, Inc).

Baseline patient characteristics, including age, race, body mass index, smoking history, diabetes mellitus, hypertension, hyperlipidemia, blood pressure (BP) medication use, statin use, history of coronary heart disease (myocardial infarction, percutaneous coronary intervention, or coronary artery bypass graft), total testosterone, and PCa-related features (T stage and Gleason score), were collected at the time of trial enrollment. Covariate normality was assessed with Shapiro-Wilk testing and visual inspection (histograms). Descriptive statistics were presented as counts (percentages) and means (SD) or medians (IQR). Lower-risk cancer was defined as low or favorable intermediate National Comprehensive Cancer Network risk, and higher-risk cancer was defined as unfavorable intermediate, high, or very high risk National Comprehensive Cancer Network risk. Comparisons between lower- and higher-risk cancer groups were assessed with *t*-test, Wilcoxon rank-sum, Pearson chi-squared, and Fisher exact tests. The independent association of higher-risk vs lower-risk cancer with CAC was assessed by logistic regression (CAC; ≥100 vs <100 AU) and with TPV by ordinal logistic regression (TPV; <250, 250-750, >750 mm^3^). Covariates were selected with purposeful variable selection (*P* < 0.25), and models were additionally adjusted for covariates that were statistically imbalanced between higher- and lower-risk cancer groups. This study was approved by the institutional review board of Emory University (Atlanta, GA).

The mean age of the sample (N = 87) was 67.6 (SD 8.2) years; 26% (N = 23) were Black, and 56% (N = 49) were prescribed statins. Clinicodemographic variables were balanced between lower-risk and higher-risk groups, aside from the higher-risk group being significantly more likely to use BP medication (*P* = 0.027). Participants with higher-risk cancer were significantly more likely to have CAC ≥100 AU (58% vs 31%, *P* = 0.038) and an increased burden of TPV (*P* = 0.033) than lower-risk participants (Table 1). Men with higher-risk cancer had significantly higher odds of having a CAC ≥100 AU than men with lower-risk cancer (OR: 8.15; 95% CI: 1.94-46.85; *P* = 0.008), independent of age, Black race, body mass index, smoking history, hyperlipidemia, BP medication use, and statin use. Regarding TPV, men with higher-risk cancer also had significantly higher odds of being in a higher TPV burden category when compared to men with lower-risk cancer (OR: 4.31; 95% CI: 1.28-16.51; *P* = 0.024), independent of age, Black race, smoking history, diabetes, hypertension, hyperlipidemia, BP medication use, and statin use.

**Table 1:** Baseline Characteristics, Coronary Artery Calcium Burden, and Coronary Total Plaque Volume by Cancer Risk Stratification

	NCCN Risk Tier		*P* Value
	Low or Favorable Intermediate Risk (n = 29)	Unfavorable Intermediate, High, or Very High Risk (n = 52)	
Age (y)	66.10 (7.19)	68.31 (8.25)	0.23
Black race (%)	6 (20.7)	14 (26.9)	0.72
Body mass index (kg/m^2^)	28.94 [27.24, 31.25]	28.60 [25.01, 32.02]	0.55
Smoking status (%)			0.71
Never	19 (65.5)	34 (65.4)	
Former	7 (24.1)	15 (28.8)	
Current	3 (10.3)	3 (5.8)	
Diabetes mellitus (%)	4 (13.8)	11 (21.2)	0.60
Hypertension (%)	17 (58.6)	41 (78.8)	0.093
Hyperlipidemia (%)	21 (72.4)	36 (69.2)	0.96
History of CHD (%)	0 (0)	0 (0)	>0.99
Blood pressure medication use (%)	12 (41.4)	36 (69.2)	0.027
Statin use (%)	18 (62.1)	27 (51.9)	0.52
Baseline testosterone, total (ng/mL)	278.8 (71.3)	270.9 (105.5)	0.72
T stage			
1	25 (89.3)	32 (64.0)	0.039
2	3 (10.7)	17 (34.0)	
3	0 (0.0)	1 (2.0)	
4	0 (0.0)	0 (0.0)	
Gleason score			<0.001
6	7 (24.1)	2 (3.8)	
7	22 (75.9)	33 (63.5)	
8-10	0 (0.0)	17 (32.7)	
Total coronary plaque volume (mm^3^) (%)			0.033
<250	22 (75.9)	25 (49.0)	
250-750	2 (6.9)	15 (29.4)	
>750	5 (17.2)	11 (21.6)	
CAC score ≥100 (AU) (%)	9 (31.0)	30 (57.7)	0.038
CAC score (AU) (%)			0.097
0	3 (10.3)	6 (11.5)	
1-99	17 (58.6)	16 (30.8)	
100-399	4 (13.8)	14 (26.9)	
≥400	5 (17.2)	16 (30.8)	

Counts (%) were provided for categorical variables, mean (SD) were provided for normally distributed continuous variables, and median [IQR] were provided for non-normally distributed continuous variables. T-tests were utilized for normally distributed continuous variables (age and total testosterone), Wilcoxon rank-sum tests were utilized for non-normally distributed continuous variables (body mass index), Pearson chi-squared tests were utilized for categorical variables with counts ≥5, and Fisher exact tests were utilized for categorical variables with counts <5.

AU = Agatston units; CAC = coronary artery calcium; CHD = coronary heart disease; NCCN = National Comprehensive Cancer Network.

We show in a diverse cohort of men with newly diagnosed, localized PCa enrolled in a prospective clinical trial that participants with higher-risk cancer had a significantly higher burden of both CAC and TPV than those with lower-risk cancer before initiation of any cancer therapy. Moreover, multivariable-adjusted regression analyses demonstrated that men with higher-risk PCa had 8-fold higher odds of having clinically significant CAC (≥100 AU) and 4-fold higher odds of having a higher TPV burden than those with lower-risk PCa. Although we are unable to assess causality, we hypothesize that more aggressive PCa phenotypes may be linked to higher atherosclerotic burden due to shared pathophysiological pathways. Biological processes such as the PI3K-AKT pathway have been shown to be associated with both more aggressive PCa as well as the promotion of atherosclerosis through endothelial cell dysfunction and lipid accumulation.^2^^,^^4^ Higher-grade PCa has furthermore been shown to exhibit more pronounced levels of systemic inflammation, with several inflammatory cytokines (ie, interleukin [IL]-1β, IL-8, and IL-10) being identified as potential mediators of this relationship, as well as in fostering pro-atherogenic environments.^2^^,^^3^ Lastly, increased exposure to oxidative stress has also been linked to more aggressive PCa and is a well-known risk factor for atherosclerosis.^5^

Limitations of our study include the small sample size and single-center recruitment, which could limit the generalizability of our findings. However, our finding of higher-risk PCa being associated with greater CAC and TPV burden––and thus higher cardiovascular risk––is important to recognize and further explore, as men with higher-risk PCa more often require ADT during their treatment course, which in itself exacerbates cardiovascular morbidity. Future studies in larger samples are needed to explore the biological basis of increased atherosclerotic burden across the PCa risk spectrum.
